# Functional cross-species conservation of guanylate-binding proteins in innate immunity

**DOI:** 10.1007/s00430-022-00736-7

**Published:** 2022-04-13

**Authors:** Luca Schelle, João Vasco Côrte-Real, Pedro José Esteves, Joana Abrantes, Hanna-Mari Baldauf

**Affiliations:** 1 Faculty of Medicine, Max Von Pettenkofer Institute and Gene Center, Virology, National Reference Center for Retroviruses, LMU München, Feodor-Lynen-Str. 23, 81377 Munich, Germany; 2 CIBIO-InBIO, Research Center in Biodiversity and Genetic Resources, University of Porto, 4485-661 Vairão, Portugal; 3 Department of Biology, Faculty of Sciences, University of Porto, 4169-007 Porto, Portugal; 4 BIOPOLIS Program in Genomics, Biodiversity and Land Planning, CIBIO, Campus de Vairão, 4485-661 Vairão, Portugal; 5 CITS-Center of Investigation in Health Technologies, CESPU, 4585-116 Gandra, Portugal

**Keywords:** Guanylate binding protein, Evolution, Innate immunity, Antiviral proteins, Cross-species conservation, Plants, Invertebrates, Mammals

## Abstract

Guanylate binding proteins (GBPs) represent an evolutionary ancient protein family widely distributed among eukaryotes. They are interferon (IFN)-inducible guanosine triphosphatases that belong to the dynamin superfamily. GBPs are known to have a major role in the cell-autonomous innate immune response against bacterial, parasitic and viral infections and are also involved in inflammasome activation. Evolutionary studies depicted that *GBPs* present a pattern of gain and loss of genes in each family with several genes pseudogenized and some genes more divergent, indicative for the birth-and-death evolution process. Most species harbor large *GBP* gene clusters encoding multiple paralogs. Previous functional studies mainly focused on mouse and human GBPs, but more data are becoming available, broadening the understanding of this multifunctional protein family. In this review, we will provide new insights and give a broad overview about GBP evolution, conservation and their roles in all studied species, including plants, invertebrates and vertebrates, revealing how far the described features of GBPs can be transferred to other species.

## Introduction

**Fig. 1:**
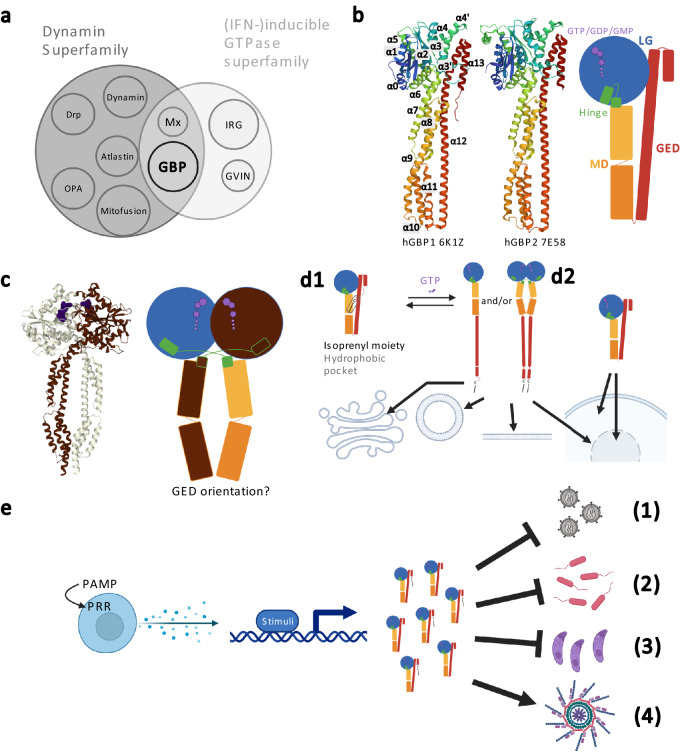
Structure and function of GBPs. **a** Depicted is the relationship of GBPs within the dynamin superfamily proteins (protein family) and (IFN)-inducible Guanosine Triphosphatases. Since this functional classification is not true for plant GBPs, IFN is put in brackets [1, 2]. **b** Depicted is the structure of GBPs: GBP comprises three main domains: the N-terminal large GTPase (LG) domain connected by a hinge region to the middle domain (MD) and the GTPase effector domain (GED) C-terminal (PDB accession numbers: 6K1Z, 7E58), the α helices are labeled [4, 10, 75, 76]. **c** The proposed model of GBP dimerization (PDB accession number: 7E5A) is given [4, 75, 76]. **d** Depicted is the proposed model for GBP localization: upon GTP/dimerization, GBPs isoprenylated at their CaaX motif anchor to membranes via released isoprenyl moiety and open state; and localize therefore to different cellular organelles (vesicle-like, plasma membrane, perinuclear membrane, Golgi*)* (D1). GBPs without isoprenylation motif or in a closed state homogeneously localize in the cytosol and few in the nucleus (D2). **e** Depicted are the proposed functions of GBP: GBPs are part of the cell-autonomous innate immune response against various bacterial, parasitic and viral infections and involved in inflammasome activation. (1) viruses; (2) bacteria; (3) parasites; (4) inflammasome activation. Figure was created with BioRender.com

GBPs are members of the dynamin superfamily (protein family) and the IFN-inducible guanosine triphosphatases. Of note, IFN inducibility is not true for GBPs in plants [1, 2] (Fig. 1a). The GBP proteins share common features and functions as outlined below:

### Structure

The information on GBPs’ structure is scarce. Indeed, until now, out of seven human GBP paralogs (hGBP1-7) only structural data for human GBP1 (hGBP1) exist [3], which has been recently extended to hGBP2/5 [4]. GBPs comprise three main domains: the large GTPase (LG) domain at the N-terminus connected by a hinge region (N-terminal part in α6 and C-terminal part in α7) to the middle domain (MD) and the GTPase effector domain (GED) at the C-terminus (Fig. 1b). The LG domain is a globular domain including five motifs: P-loop (G1), switch I (G2), switch II (G3), (N/T) KxD motif (G4) and the guanine cap (G5). These motifs are involved in GTP binding/orientation, Mg^2+^ cofactor finding and GTP/GDP hydrolysis [2, 4, 5]. The MD is an α-helical elongated domain (α7-11) comprising two three-helix bundles (α9 is shared). The GED is an α-helical elongated domain (α12-13) which, in nucleotide free state, is folded onto LG and MD.

### Dimerization and polymerization

Recently, it has been described that hGBPs probably share a conserved dimerization mode between paralogs [4]. Upon GTP binding and LG:LG interface building, the GBP structurally rearranges to an open state driven by GTPase hydrolysis cycles. Kinetically delayed, the MD domain rearranges beneath the LG domain of the second GBP. Hereby, the hinge regions cross each other and form a closed dimeric state, which is further stabilized by the MD interface (Fig. 1c) [4, 6–9]. For hGBP1, farnesylation and GTP-dependent polymerization have been observed, but the exact function remains unclear [9].

Based on current knowledge it may be hypothesized that the conserved closed dimeric state represents the actual “active” form of specific GBPs’ innate immunity-related functions but not all functions have to be solely related to dimerization [4].

### Localization and membrane anchoring

hGBP1/2/5 harbor a CaaX motif at the C-terminus of the GED, which serves as a signal for in vivo isoprenylation (GBP1: farnesylated; GBP2/5: geranylgeranylated) and membrane anchoring. In a closed monomeric state hGBP1/2/5 localize homogenously distributed in the cytoplasm. Further, the isoprenyl moiety is buried in a hydrophobic pocket between GED (α12) and MD (α9) [2, 4, 5, 10]. Favored by the described GTP binding/hydrolysis and intra-dimeric interactions, the buried isoprenyl moiety is released from the hydrophobic pocket leading to a rearrangement into an open state. Subsequently, the released isoprenyl moiety is the determinant for membrane anchoring and, consequently, for the localization to the membranes at the cytosolic face of cellular compartments (Fig. 1d) (hGBP1: vesicle-like, plasma membrane; hGBP2: perinuclear membrane; hGBP5: Golgi). [4, 6, 8, 10]. Whereas the non-isoprenylated hGBP3/4 stay homogeneously localized in the cytosol or sometimes localized in the nucleus (Fig. 1d) [6, 11], hGBP4/6 can also be found to colocalize with vesicle-like structures without being isoprenylated [12]. It has also been described that homo- and heterodimerization influence localization [6, 11] but details are not yet clear.

### GBP functions and roles in innate immunity

The expression of *GBPs* is triggered by inflammatory signals. The most potent stimuli for expression are interferons (IFN) due to IFN-stimulated response elements in the 5′ cis regulatory region of the *hGBP* genes. GBPs are among the most upregulated genes upon IFNγ stimulation. Especially hGBP1/5 expression is upregulated by up to two to three orders of magnitudes [13]. GBPs can be further stimulated by interleukins (ILs) and tumor necrosis factors (TNFs), but to a much lesser extent (reviewed in [13]).

The IFN-inducibility hints to some functions of GBPs. They are part of the cell-autonomous innate immune response against various pathogens and, in this context, are involved in canonical and non-canonical inflammasome activation. They respond to various intracellular bacteria, mostly gram negative, but also gram positive, as well as parasites (e.g., *Shigella flexneri*, *Salmonella enterica*, *Salmonella typhimurium*, *Legionella pneumophila*, *Francisella novicida*, *Chlamydia trachomatis*, *Listeria monocytogenes*, *Mycobacterium bovis*, *Leishmania donovani* and *Toxoplasma gondii*) (reviewed in [13, 14]). Moreover, GBPs inhibit viral infections such as vesicular stomatitis virus (VSV), classical swine fever virus (CSFV), murine norovirus-1 (MNV-1), Newcastle disease virus (NDV), encephalomyocarditis virus (EMCV), dengue virus (DENV), herpes simplex virus type 1 (HSV-1), Kaposi’s sarcoma-associated herpesvirus (KSHV), hepatitis E virus (HEV), hepatitis C virus (HCV), influenza A virus (IAV), human immunodeficiency virus (HIV) and respiratory syncytial virus (RSV) (Fig. 1e) (reviewed in [14, 15]).

Taken together, GBPs have been considered as major players in the host innate immunity by providing defense against a broad range of invading pathogens.

## GBP evolution and conservation

The origin and evolution of *GBPs* have been analyzed only recently with most of the evolutionary history of *GBPs* still unclear [16–19]. *GBPs* originated from a common ancestor and belong to the multigene family of the large dynamin superfamily [20]. *GBPs* can be found in a broad range of organisms from plants to humans [18]. The presence of *GBPs* in plants species like *Arabidopsis thaliana*, *Oryza sativa* and *Solanum lycopersicum* indicates that *GBPs* are active in organisms that do not present migratory immune cells and an IFN-inducible immune system [18].

In mammals, *GBP* genes are usually organized in tandem on the same chromosome [19, 20]; however, in some rodents, like *Mus* and *Rattus norvegicus*, the *Gbps* are located on two gene clusters on different chromosomes [16]. In addition, in zebrafish and frogs, *gbp* genes are found in three small genomic islands [13]. Plants also have a variation regarding the number of *GBPL* (GBP-like) genes present in their genome, for example, *Oryza sativa* has three orthologs, while in *Arabidopsis thaliana* and *Zea mays* seven *GBPL* are encoded in their genome [18]. Altogether, this suggests that independent duplication events contributed to *GBP* diversity across plant and animal kingdoms [18]. Moreover, since *GBPs* are a multigene family that belongs to the immune system, it follows the birth-and-death process of evolution [21]. This results in some genes being either deleted or maintained in the genome. When maintained, the genes can acquire a new function (neofunctionalization), split functions (subfunctionalization) or even lose function and become pseudogenes [17, 22]. For example, *GBP3* gene appears to have emerged only in Simiiformes through a duplication of *GBP1* and gained a new function being responsible for the regulation of caspase-4 activation (Table 1) [23]. As for *GBP7*, it most likely emerged from a duplication event of *GBP4* and seems to be only present in primates (Table 1) [17].

**Table 1:** General overview of GBP genes in Primates and Muroids

	Primates		Muroids		
	New world monkeys and great apes	Old world monkeys	*Muridae*	*Cricetidae*	*Mus musculus*
*GBP1*	+	+	−	−	−
*GBP2*	+	+	+	+	+
*GBP3*	+/*ψ*	+	−	−	−
*GBP4*	+	−	−	−	−
*GBP5*	+	−	+	+	+
*GBP6*	+	+	+	+	+
*GBP7*	+/*φ*	+	−	−	−
*Gbpa*	−	−	+/*ω*	+/*ω*	−
*Gbpb*	−	−	+/*ω*	+/*ω*	−
*Gbpc*	−	−	+/*ω*	+/*ω*	−
*Gbpd*	−	−	+/*ω*	+/*ω*	+

+, present; −, not present; *ψ*, exclusive to Simiiformes; *φ*, exclusive to Primates; *ω*, exclusive to Muroids

*GBP4* and *GBP5* seem to have been deleted from the genomes of Old-World monkeys and the lack of *GBP5* orthologs might explain the HIV-2 transmission susceptibility in these primates since *GBP5* inhibits HIV-2 infection [13, 17].

Some *GBP* orthologs are not present in different species, while others might be exclusive to specific orders. According to phylogenetic analyses it appears that primate *GBP1, GBP3* and *GBP7* are absent from muroid genomes (Table 1) [16]. This further indicates that the nomenclature of muroids *Gbps* has been incorrect and functional studies of these *Gbps* might have led to misleading results [16]. Following an evolutionary study in muroids, *Gbp2*, *Gbp5* and *Gbp6* have been found to be orthologs to their primate counterparts [16].

*Gbp2* is found in every family of muroids and duplication events occurred in all genera except in *Rattus*. *Gbp5* presents only one copy in each species of muroids, similar to primates. Maintenance of *Gbp2* and *Gbp5* in the muroid genomes supports the importance of these two genes for the host immune system [24, 25].

Phylogenetic analyses in Muroidea and Cricetidae indicate the presence of four *Gbps* that are exclusive to these taxa (*Gbpa, b, c* and *d*) (Table 1) [16]. The *Gbpa* and *Gbpb* groups are mainly composed of *Gbps* previously classified in public databases (NCBI and Ensembl) as *Gbp1* [16]. Phylogenetically, they are not similar to *hGBP1*. Interestingly, these genes are not present in *Mus musculus* [16]. The function of these genes has yet to be determined, but the study of the sequences and the 3D structure of the proteins may provide hints on their function. *Gbpc* is only present in three species, being absent in *Mus musculus*, but its function is also not known. Considering the *Gbpd* group, three main groups emerged and are present in all species of muroids indicating a possible duplication in the common ancestor of Muridae and Cricetidae (Table 1) [16]. The *Mus musculus* classified as *Gbpd1* [16]*,* previously annotated in NCBI and Ensembl as *Gbp7,* appears to be a cellular host dependency factor for IAV replication [26].

*Gbp6* cluster is present in most Muridae and Cricetidae species, and in *Mus musculus* and *M. caroli*, an expansion of this gene has observed, with *Mus musculus* presenting six copies and *Mus caroli* four. This expansion might be explained as a compensation mechanism due to the lack of *Gbpa*, *b* and *c* in these two species [16].

The evolutionary history of the *GBP* multigene family is complex and dynamic with duplication (*Gbp2* and *Gbp6* in several species), deletion (*Gbpa*, *b* and *c* in *Mus musculus*; Table 1) and neofunctionalization (*GBP3* in primates) of genes, in line with the proposed birth-and-death mode of evolution [17]. In each mammalian family, the different evolutionary histories open new research opportunities to study the evolution and function of *GBPs*, which should be conducted in a more holistic approach.

## GBP functions in plants, invertebrates and vertebrates

### GBPs in plants

**Fig. 2:**
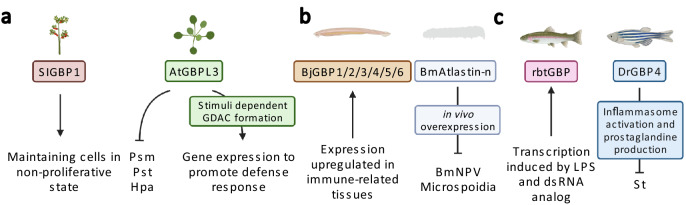
Plant, invertebrate and teleost GBPs in innate immunity. **a** Plants: AtGBPL3 confers plant defense against Psm, Pst and Hpa. Further, stimuli-dependent formation of GDACs reprogram host gene expression to promote defense response. SIGBP1 maintains cells in a non-proliferative state. **b** Invertebrates: BjGBPs expression is upregulated in immune-related tissues. BmAtlastin-n inhibits in vitro and in vivo replication of BmNPV and microsporidia. **c** Teleosts: The transcription of rbtGBP is induced by LPS and dsRNA analogs. DrGBP4 supports clearance of *St* infections via inflammasome activation and prostaglandine production. Abbreviations: *Arabidopsis thaliana* (At), *Pseudomonas syringae* pv. *maculicola* (Psm), *Pseudomonas syringae* pv. Tomato (Pst), *Hyaloperonospora arabidopsidis* (Hpa), GBPL defense-activated condensates (GDAC), *Solanum lycopersicum* (Sl), *Branchiostoma japonicum* (Bj), *Bombyx mori* (Bm), double-stranded (ds), nucleopolyhedrovirus (NPV), rainbow trout (rbt), lipopolysaccharide (LPS), *Danio rerio* (Dr), *Salmonella typhimurium* (St), mouse (m), *Listeria monocytogenes* (Lm), *Mycobacterium bovis* (Mb), vesicular stomatitis virus (VSV), encephalomyocarditis virus (EMCV). Figure was created with BioRender.com

GBP-like proteins seem to be widely distributed as they even exist in plants. Plants solely rely on innate immune mechanisms to resist against phytopathogens (reviewed in [27, 28]). GBPs are poorly characterized in plants, but first results have been obtained in recent years. Indeed, tomato (*Solanum lycopersicum*) GBP homolog, SIGBP1, has been reported to be involved in fruit tissue differentiation by maintaining cells in a non-proliferative state [29] (Fig. 2A). First comparisons of the modeled structure of *Arabidopsis* GBP-like (AtGBPL) to hGBP1 crystal structure revealed a similar architecture. AtGBPL1/3 seem to comprise an intrinsically disordered region (IDR) at the C-terminus instead of an isoprenylation motif [18]. Functional studies with AtGBPL1/2/3 have revealed the roles of AtGBP1 (negative allosteric regulator of AtGBP3) and AtGBP3 in host defense. Indeed, they confer resistance to phytopatogens such as *Pseudomonas syringae* pv. *maculicola* (Psm), *Pseudomonas syringae* pv. Tomato (Pst) and *Hyaloperonospora arabidopsidis* (Hpa). Upon salicylic acid, pipecolic acid or phytopathogen activation, AtGBP3 condensates to unique membraneless organelles, termed GBPL defense-activated condensates (GDACs), within the nucleus, binding defense-gene promotors and recruiting transcriptional coactivators. This, in turn, reprograms the host gene expression to promote host defense responses (Fig. 2a). GDACs have also been observed in tomato and maize, which could hint for a conserved mechanism in plants [15]. Since phytohormone salicylic acid biosynthesis is also promoted by plant viruses (reviewed in [30]), it seems possible that AtGBPLs also might be involved in antiviral response, but this hypothesis needs to be proven.

In summary, GBP-dependent innate immunity processes are present in plants and animals and, thus, probably exist already over a longer period of time.

### GBPs in invertebrates

The function/presence of GBPs in invertebrates is still unclear. Indeed, in silico analyses have revealed that non-vertebrate species harbor *GBP-*like genes, but not all of them seem to be completely lacking them [13, 20]. If this is due to a low genome coverage or, in fact, if these genes are not present still needs further clarification. In amphioxus (*Branchiostoma japonicum*), expression of *GBPs* is upregulated in immune-related tissues [20] (Fig. 2b), which could indicate their involvement in innate host defense.

Recently, the BmAtlastin-n protein of silkworm (*Bombyx mori*) has been suggested to be part of the GBP family [31] due to the lack of the typical atlasin transmembrane domain [32] and similarity in the GTPase domain [31]. Transgenic silk worms overexpressing BmAtlastin-n have shown in vitro and in vivo inhibition of viral reproduction capacity of *Bombyx mori* nucleopolyhedrovirus (BmNPV), a virus causing nuclear polyhedrosis [32]. The mechanism of viral inhibition is elusive, but it seems to correlate with the reduction of VP39 (capsid protein from late baculovirus gene) expression levels (mRNA and protein) [32]. Furthermore, it also enhances in vivo resistance against the obligate intracellular parasite microsporidia. Therefore, BmAtlastin-n seems to protect from intracellular infections caused by more than one pathogen (Fig. 2b), similar to other GBPs.

Why some invertebrates harbor GBPs in their genome and others seem to have lost them remains an open question requiring further investigations. Since atlastins and GBPs are closely related, it raises the question if in invertebrates without GBP homologs atlastins may have adopted some of their defense functions or if their common ancestor already possessed anti-pathogenic functions.

### Gbps in teleosts

Studies regarding Gbps in teleosts are scarce. The first characterization of Gbps in fish has been in 2006 by Robertsen and colleagues [33], while mammalian GBPs have been described since 1983 [34]. The Gbp found in rainbow trouts (rbtGBP) appears to have a similar structure as hGBP1 with similar domains and a CaaX motif at the C-terminus, responsible for isoprenylation. Moreover, the most conserved region is the N-terminal surrounding GTP-binding region (amino acid 6–278) [33], while the C-terminal region is 43 amino acids longer compared to the human counterpart [33]. rbtGbp shares 41 to 47% amino acid sequence identity with mammalian GBPs. Interestingly, the region encompassing the GTP-binding motifs shares 67% identity with mammals. However, the C-terminus has only 37% identity with the mammalian GBPs [33]. The transcription level of rbtGBP is upregulated by lipopolysaccharide (LPS) and polyinosinic polycytidylic acid (poly I:C, double-stranded RNA analog) [43] (Fig. 2c). This may hint for an involvement of rbtGBP in innate immunity against bacteria and RNA viruses. Zebrafish Gbp is similar in length to the rbtGbp, but lacks a CaaX motif at the end of the C-terminus [33]. Nevertheless, DrGbp may play a role in the innate immunity against bacterial infections since DrGbp4 is involved in inflammasome activation and clearance of *Salmonella typhimurium* (St) infections [35].

In *Danio rerio*, eight Gbps have been found, with two Gbps being studied until now, Gbp1 and Gbp4. The nomenclature of *gbps* in fish is probably inaccurate since they do not cluster with their human counterparts, similar to the observations in muroids [26]. DrGbp1 contains an N-terminal GTPase domain and a helical C-terminal domain similar to mammalian GBPs [36]. DrGbp4 has a similar architecture as DrGBP1 with an additional C-terminal caspase recognition domain (CARD) and shares 53% identity with hGBP5 [35]. DrGbp4 is an IFNγ-induced GTPase, similar to mammalian GBPs. It is expressed in neutrophils, but in macrophages expression levels were hardly detected [35]. Tyrkalska and colleagues have demonstrated the paramount role of Gbp4 in bacterial clearance, being crucial for the biosynthesis of prostaglandins via an inflammasome-dependent pathway to clear *St* bacterial infection [35]. The GTPase activity of Gbp4 is crucial for caspase-1 activity, inflammasome activation and resistance to infection by *St* bacterial infection [35]. Indeed, Gbp4-deficient fish have a negatively affected caspase-1 activity and display increased susceptibility to *St* infections compared to fish with wildtype Gbp4. Interestingly, when Gbp4-deficient fish are trans-complemented with mouse *Gbp5*, *St* susceptibility decreases and caspase-1 defects are rescued [35]. Additionally, DrGbp4 regulates the expression of WD repeat domain 90 (WDR90), which is a component of the NOD-like receptor with CARD domain 4 inflammasome and is responsible for the conformational change needed for its activation [37] (Fig. 2c). Altogether, in fish, Gbps appear to have also an important role in the innate immune system, especially for bacterial infection. However, more studies are needed to further understand the functions of Gbps in teleost.

### GBPs in mammals

Several studies have already been performed to understand the functions of GBPs in humans and, at some extent, in rodents and few further mammals; however, in general, the function of the majority of the mammalian GBPs remains unclear.

**Fig. 3:**
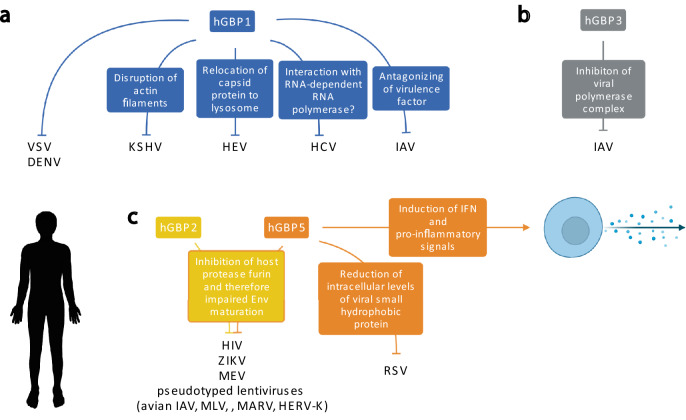
Antiviral activity and underlying mechanisms of hGBPs. **a** hGBP1: Restriction of VSV and DENV by unknown mechanism. Restriction of KSHV by disruption of actin filaments, resulting in impaired transport. Restriction of HEV by relocation of the capsid protein to the lysosome. Interaction with RNA-dependent RNA polymerase (NS5B) of HCV might explain restriction. For IAV, the NS1 virulence factor is antagonized by hGBP1. **b** hGBP3: Inhibition of the viral polymerase complex of IAV. **c** hGBP2/5: Interference with host protease furin, which impairs HIV glycoprotein maturation, resulting in a decreased infectivity of released viral particles. Same has been described for Zika virus (ZIKV), measles virus (MEV) and lentiviral particles pseudotyped with various envelope glycoproteins (avian IAV, murine leukemia virus (MLV), Marburg virus (MARV) and human endogenous retrovirus K (HERV-K) Env glycoproteins. hGBP5 further restricts the replication of RSV by reducing intracellular levels of the viral small hydrophobic protein. hGBP5 has also a more general role in innate immunity as it can induce enhanced production of IFN and proinflammatory signals. Abbreviations: human (h), vesicular stomatitis virus (VSV), dengue virus (DENV), Kaposi’s sarcoma-associated herpesvirus (KSHV), hepatitis E virus (HEV), hepatitis C virus (HCV), influenza A virus (IAV), human immunodeficiency virus (HIV), murine leukemia virus (MLV), Zika virus (ZIKV), measles virus (MEV), Marburg virus (MARV), human endogenous retrovirus K (HERV-K), respiratory syncytial virus (RSV), Interferon (IFN). Figure was created with BioRender.com

Since we would like to emphasize in this review the roles of non-human GBPs, we only shortly point out the antiviral activity of hGBPs. Needless to say their activity against bacteria and parasites are not less important, they have been recently reviewed in detail in [13, 14]. hGBP1/2/3/5 are known to be involved in restriction of viruses, employing thereby various mechanisms and targeting different steps in their life cycle. Yet, the underlying mechanisms remain elusive for specific viruses [14, 15]. hGBP1 employs several mechanisms to restrict viruses (Fig. 3a). For KSHV, the transport of the viral capsid to the nucleus is hampered by disruption of the actin filaments by hGBP1 [38]. HEV is inhibited through the relocation of the capsid protein by hGBP1 to the lysosome [39]. For HCV, the observed interaction with RNA-dependent RNA polymerase NS5B could be a possible explanation for the viral restriction [40]. In the case of IAV, the NS1 virulence factor is antagonized by hGBP1 [41]. For other viruses (e.g., VSV, DENV) the mode of action for their inhibition by GBP1 remains unknown [42, 43]. hGBP1 may employ similar mechanisms as mentioned above to inhibit the other viruses but also other mechanisms are conceivable. hGBP3 has only now been identified to play a role in IAV infection by inhibiting the viral polymerase complex [44] (Fig. 3b). GBP2/5 interfere with the host protease furin, which impairs HIV glycoprotein maturation resulting in a decreased infectivity of released viral particles [12, 45]. This has been also observed for Zika virus (ZIKV), measles virus (MEV) and lentiviral particles pseudotyped with various envelope glycoproteins (avian IAV, murine leukemia virus (MLV), Marburg virus (MARV) and human endogenous retrovirus K (HERV-K)) [12, 45, 46]. GBP5 further restricts the replication of RSV by reducing intracellular levels of the viral small hydrophobic protein [47]. Thus, GBP5 is generally involved in innate immunity as it can induce enhanced production of IFN and proinflammatory signals [48] (Fig. 3c).

Five pig (p) GBPs are described in literature. Based on NCBI, *Sus scrofa* has 7 *GBPs* in one gene cluster on chromosome 4 (accession numbers: NM_001128473.1, NM_001128474.1, XM_005663706.3, XM_021090310.1, XM_013997408.2, XM_021090315.1, XM_005663708.3). Only pGBP1/2 have been characterized on protein level. They share a conserved N-terminal GTPase domain and a C-terminal CaaX motive similar to other mammalian GBPs [49]. Pig GBP research is limited to pathogens especially affecting the global swine industry: the respiratory syndrome virus (PRRSV) and classical swine fever virus (CSFV) [50]. CSFV replication is potently inhibited by pGBP1 via its GTPase activity. pGBP1 mainly acts in the early phase of viral replication by inhibiting the translation efficiency of the internal ribosome entry site (IRES). Notably, CSFV NS5A protein counteracts pGBP1’s antiviral activity by inhibition of the GTPase activity [50]. For PRRSV, a quantitative trait locus (QTL) on *Sus scrofa* chromosome (SSC) 4 has been identified being beneficial for controlling infection. The characterization of this QTL revealed that it contains inter alia pGBP1/2/4/5/6 and that the QTL is associated with resistance to PRRSV infection. Furthermore, pGBP1/5/6 lead to a reduction of PRRSV viral loads in vivo in pigs [51–54]. Yet, the underlying mechanisms remain elusive.

*Tupaia* has 5 copies of *GBPs* in one gene cluster similar to humans, while most rodents present two gene clusters [19, 55]. Also similar to human and mouse GBPs, the coding region of *Tupaia GBPs* (*tGBPs*) ranges from 1733 to 1884 bp and the molecular weight of the proteins is between 67 to 72kD [55]. Most of the conserved motifs are present, particularly in the N-terminus where the GTPase domain is located. As expected, the C-terminus shares low sequence identity among the different groups. Phylogenetically, the sequences of *tGBP* genes are clustered with the h*GBP* genes, which indicates that the *Tupaia* genes are human orthologs [55]. Only in *tGBP1*, *tGBP2* and *tGBP5* a CaaX motif is present as in humans and mice [13, 56, 57]. This motif allows isoprenylation and consequently the anchorage to membranous organelles, enabling the destruction of pathogen-containing vacuoles, mainly bacterial pathogens, which exposes the pathogen to the host [15, 58–60].

**Fig. 4:**
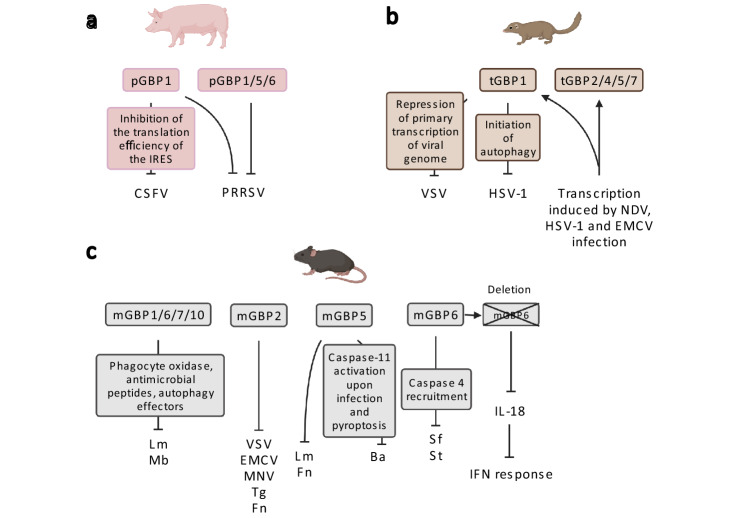
Non-human mammalian GBPs in innate immunity. **a** Pig: pGBP1 inhibits CSFV by inhibition of the translation efficiency of the IRES. pGBP1/5/6 reduce viral loads of PRRSV in vivo in pigs via unknown mechanisms. **b** Tupaia: The transcription of tGBP1/2/4/5/7 is upregulated upon VSV, HSV-1 and NDV infection. tGBP1 restricts VSV by repression of primary transcription of the viral genome. tGBP1 further restricts HSV-1 via initiation of autophagy. **c** Mouse: mGBP1/6/7/10 restrict Lm and Mb combined via phagocyte oxidase, antimicrobial peptides, and autophagy effectors. mGBP2 displays restriction towards various viral, bacterial and parasitic pathogens (VSV, EMCV, MNV, Tg, Fn). mGBP5 restricts Lm and Fn. mGBP5 further inhibits Ba through Caspase-11 activation and pyroptosis. mGBP6 restricts Sf and *St* via Caspase-4 recruitment. Deletion of mGBP6 leads to reduced IFN response. Abbreviations: pig (p), respiratory syndrome virus (PRRSV), classical swine fever virus (CSFV), internal ribosome entry site (IRES), *Tupaia* (t), vesicular stomatitis virus (VSV), Newcastle disease virus (NDV), type 1 herpes simplex virus (HSV-1), encephalomyocarditis virus (EMCV), mouse (m) *Listeria monocytogenes* (Lm), *Mycobacterium bovis* (Mb), murine norovirus (MNV), *Toxoplasma gondii* (Tg), *Francisella novicida* (Fn), *Bacillus abortus* (Ba), *Shigella flexneri* (Sf), *Salmonella typhimurium* (St), interleukin (IL), interferon (IFN). Figure was created with BioRender.com

When acute signaling is absent, hGBPs are expressed at low to medium levels in immune cells, lung, liver, kidney, brain and skin [13, 61]. tGBPs are also ubiquitously expressed at low levels in heart, spleen, kidneys, intestines, liver, lung and brain [55]. Human, mouse and *Tupaia GBPs* are strongly induced by IFN [19, 55, 62, 63] and *Tupaia* mRNA levels of *GBPs* are increased after RNA virus infections of primary renal cells such as Newcastle disease virus (NDV) and encephalomyocarditis virus (EMCV), and DNA virus type 1 herpes simplex virus (HSV-1) [55] (Fig. 4b).

As outlined above, hGBP1 is the most studied GBP, it has been described to have antiviral activity against a broad range of viruses [38, 40, 42, 64]. In *Tupaia*, tGBP1 is the only GBP from the five tGBPs that displays antiviral activity against VSV and HSV-1. It significantly represses the primary transcription of VSV viral genomes, but only presents a rather moderate effect against HSV-1 [55]. For VSV-G, tGBP1 restricts the viral genomic transcription in the cytoplasm by competitively binding to the VSV-N subunit [55]. The moderate HSV-1 inhibition by tGBP1 is tSTING-dependent, promoting tSTING-mediated autophagy, but the mechanism remains unclear. The authors speculated that autophagy could clear pathogens and DNA from the cytoplasm [65].

All tGBPs are upregulated through different viral infections, which suggests they may play a role in antiviral immunity (Fig. 4B). Yet, it is unclear how they inhibit viral replication, infectivity and proliferation [55]. The other four tGBPs need to be further investigated as *Tupaia* is becoming a recognized animal model to study human diseases (e.g., metabolic, brain aging, neurological, psychiatric and cancer) due to its closer relationship to humans than rodents [55] and also to its susceptibility to a wide range of human pathogens (HCV, HSV and SARS-CoV-2) [55, 64, 66, 67].

Murine GBP functions are the second most studied after human GBPs. As previously described, they are important for the host defense against pathogens and inflammasome activation. mGBP2 antiviral activity has been first described in 2005, revealing inhibition of VSV and EMCV replication [68]. EMCV replication inhibition requires GTPase activity of mGBP2, unlike the inhibition of VSV replication [68]. Murine norovirus (MNV) replication is inhibited when *mGBP2* is expressed in mouse macrophages. The N-terminus of mGBP2 is crucial for anti-MNV activity since only GBP2 mutants that express the G domain and the GM domain inhibit viral replication at RNA and protein level, M domain alone and the remaining domains did not present anti-MNV activities [69]. hGBP2 and hGBP5 have been described to exert a broad antiviral activity against Zika virus, measles, HIV-1 and influenza A virus by reducing their replication and also impairing furin-mediated processing of envelope glycoproteins leading to a decrease in infectivity [12, 45]. Despite the phylogenetic analyses and the conserved function of GBPs, the antiviral functions of mGBP2 and mGBP5 are yet to be fully disclosed and further studies are needed.

Additional studies demonstrate that *mGbp2* knockout increases susceptibility to infections with *Toxoplasma gondii* and *Francisella novicida*; yet mGBP2 did protect against infections with *Listeria monocytogenes* [24, 70]. mGBP5 also provides host defense against bacterial infections such as *L. monocytogenes* and *F. novicida* [24, 25]. In mouse macrophages, mGBP5 mediates caspase-11 activation and pyroptosis upon *Bacillus abortus* infection; knockdown of *mGbp5* decreased IL-1β concentrations and, expectedly, bacterial count in macrophages is increased [71, 72].

For the newly classified *Gbp6*, previously designated *Gbp4* in *Mus musculus* [16], Wandel and colleagues demonstrated its importance in caspase-4 recruitment, with the depletion of *Gbp4* in cells leading to the inability of processing and releasing IL-18 during *Shigella flexneri* and *Salmonella typhimurium* infection [23], confirming that GBPs are crucial for inflammasome activation and bacterial clearance. Most studies have focused on the individual function of each mGBP; however, the combined function of GBPs is starting to be addressed. Indeed, *en bloc* knockout of *mGBPs* located on chromosome 3 leads to reduced release of IL-18 and IL-1β via canonical NLRP3 and AIM2 inflammasomes, which is needed for IFN-γ production and host defense against bacteria, ultimately increasing susceptibility of infection [24, 73]. Moreover, it has been described that mGBP1, mGBP6, mGBP7 and mGBP10 are paramount to hamper virulent strains of *L. monocytogenes* and *M. bovis* in mouse involving phagocyte oxidase, antimicrobial peptides and autophagy effectors [63]. Silencing m*Gbps* with siRNAs has indicated that the protective effects of mGBPs operate in a collaborative way, since the combination of siRNAs decreased the killing ability via IFN-γ [63] (Fig. 4c).

Curiously, the expression of all *Gbps* located on chromosome 3 have displayed a beneficial interaction which limited acute inflammatory bone loss since *Gbp*^*Chr3−/−*^ mouse cells exhibit increased bone loss compared to wildtype [74].

## Concluding remarks

GBPs exist in a variety of eukaryotic organisms ranging from plants to animal kingdoms. Despite playing an important role in the innate immunity, the evolutionary history of *GBPs* as a multigene family is not yet fully disclosed. The immune system is continuously challenged by a broad range of intracellular pathogens, which leads to a complex evolution of the innate immunity genes. In each family, the number of *GBPs* varies, presenting several events of duplication, pseudogenization and deletion. Human and mouse GBPs have been characterized in more detail, but mostly restricted to GBPs 1/2/5. Yet, even for those, many functions remain undetermined as GBPs seem to be involved in a complicated cellular network. In this review, we provide insights on the maintenance of GBPs basal functions, like resistance to pathogens (viral, bacterial and parasitic); however, the detailed mechanisms and networks among species have not yet been sufficiently characterized. Therefore, studies on GBPs including more species may be beneficial to further understand the complex GBP network and their functions. It will be also crucial to understand the differences within the *GBP* gene clusters even in closely related species.
